# Orally Active Antischistosomal Early Leads Identified from the Open Access Malaria Box

**DOI:** 10.1371/journal.pntd.0002610

**Published:** 2014-01-09

**Authors:** Katrin Ingram-Sieber, Noemi Cowan, Gordana Panic, Mireille Vargas, Nuha R. Mansour, Quentin D. Bickle, Timothy N. C. Wells, Thomas Spangenberg, Jennifer Keiser

**Affiliations:** 1 Department of Medical Parasitology and Infection Biology, Swiss Tropical and Public Health Institute, Basel, Switzerland; 2 University of Basel, Basel, Switzerland; 3 Department of Infection and Immunity, London School of Hygiene and Tropical Medicine, London, United Kingdom; 4 Medicines for Malaria Venture, Geneva, Switzerland; McGill University, Canada

## Abstract

**Background:**

Worldwide hundreds of millions of schistosomiasis patients rely on treatment with a single drug, praziquantel. Therapeutic limitations and the threat of praziquantel resistance underline the need to discover and develop next generation drugs.

**Methodology:**

We studied the antischistosomal properties of the Medicines for Malaria Venture (MMV) malaria box containing 200 diverse drug-like and 200 probe-like compounds with confirmed *in vitro* activity against *Plasmodium falciparum*. Compounds were tested against schistosomula and adult *Schistosoma mansoni in vitro*. Based on *in vitro* performance, available pharmacokinetic profiles and toxicity data, selected compounds were investigated *in vivo*.

**Principal Findings:**

Promising antischistosomal activity (IC_50_: 1.4–9.5 µM) was observed for 34 compounds against schistosomula. Three compounds presented IC_50_ values between 0.8 and 1.3 µM against adult *S. mansoni*. Two promising early leads were identified, namely a N,N′-diarylurea and a 2,3-dianilinoquinoxaline. Treatment of *S. mansoni* infected mice with a single oral 400 mg/kg dose of these drugs resulted in significant worm burden reductions of 52.5% and 40.8%, respectively.

**Conclusions/Significance:**

The two candidates identified by investigating the MMV malaria box are characterized by good pharmacokinetic profiles, low cytotoxic potential and easy chemistry and therefore offer an excellent starting point for antischistosomal drug discovery and development.

## Author Summary

To date, praziquantel is the only available drug for the treatment of the tropical neglected disease schistosomiasis and is widely used in morbidity control programs. To discover new chemical scaffolds for the treatment of schistosomiasis, we investigated the Medicines for Malaria Venture malaria box containing 200 diverse drug-like and 200 probe-like compounds with known antimalarial activity against *Schistosoma mansoni*. Compounds were first investigated on the larval stage of *S. mansoni*, followed by testing against adult worms *in vitro* and by *in vivo* studies of lead candidates. We identified two entirely new chemical scaffolds: the N,N′-diarylurea and 2,3-dianilinoquinoxaline derivatives with antischistosomal *in vitro* activity in the sub micromolar range and significant activity in the mouse model. Since both compounds offer a good pharmacokinetic profile, low cytotoxic potential and easy chemistry, structure-activity relationship studies should be launched.

## Introduction

With hundreds of millions of people living at risk of infection and 207 million people infected with schistosomes worldwide, schistosomiasis is one of the most devastating parasitic diseases in tropical countries and remains a major public health problem, especially in Sub-Saharan Africa [1], [2]. *Schistosoma haematobium*, *S. japonicum* and *S. mansoni* are the main schistosome species, responsible for the largest number of infections [3], [4].

A major cornerstone of schistosomiasis control programs is the treatment of at risk populations with praziquantel, with the aim of controlling morbidity and preventing associated mortality [5]–[7]. Praziquantel, discovered in the 1970's, is the only drug available for the treatment of schistosomiasis [7]–[9].

Despite many benefits of praziquantel, most notably its high efficacy and excellent tolerability, the drug has major drawbacks, most importantly its inefficacy against juvenile schistosomes [10], [11]. Furthermore the increasing administration of praziquantel to millions of people annually [12] results in high drug pressure, and thus drug-resistant parasites are likely to evolve [13].

These facts underline the urgent need to discover and develop the next generation of antischistosomals. Only a few compounds are presently being studied in the preclinical phase [14]–[17] and none of the candidates evaluated in clinical trials in the past years (e.g. mefloquine [18] or the artemisinins [19]) (Figure S1) met the target product profile for a novel antischistosomal drug [20].

Interestingly many of the chemical scaffolds that revealed promising activity against schistosomes had their origin in antimalarial research and discovery [21]. The blood-feeding characteristic that both parasites have in common forms the basis for the dual antimalarial and antischistosomal activity of drugs interfering with the parasites' hemoglobin degradation pathway [22], [23].

The aim of the present study was to investigate the antischistosomal properties of the Medicines for Malaria Venture (MMV) malaria box containing 200 diverse drug-like compounds (which fit in the “Lipinski space” or rule of five), as a starting point for oral drug discovery and development, and 200 diverse probe-like compounds (no filters applied). Note that all of the compounds in the box have confirmed activity against the blood-stage of *Plasmodium falciparum in vitro* and are commercially available [24]. Studying this diverse set of molecules might reveal an entirely new chemical scaffold for antischistosomal drug discovery and therefore fill up the empty antischistosomal drug pipeline.

At the Swiss Tropical and Public Health Institute (Swiss TPH), drugs were first studied against schistosomula *in vitro* followed by a re-evaluation of successful hits on adult *S. mansoni*. In parallel all the drugs were independently tested at the London School of Hygiene and Tropical Medicine (LSHTM) in an *in vitro* adult worm assay. Possible class effects and structure-activity relationships are discussed. The onset of action and IC_50_/IC_90_ ratios were studied. Based on *in vitro* performance and available pharmacokinetic profiles as well as toxicity data, selected compounds were investigated *in vivo*.

## Methods

### Drugs and Media

The MMV Box [24], containing 400 compounds as stock solutions dissolved in dimethylsulfoxide (DMSO), concentration 10 mM, was kindly provided by MMV/SCYNEXIS, Inc. (Geneva, Switzerland; Durham, USA). For the *in vitro* studies on adult worms at the Swiss TPH and the *in vivo* studies in mice 5–100 mg of **1**: MMV000963, **2**: MMV665852, **3**: MMV665807, **4**: MMV019555, **5**: MMV019918, **6**: MMV000445, **7**: MMV019780, **8**: MMV665927, **9**: MMV665941, **10**: MMV000634, **11**: MMV665830, **12**: MMV666054, **13**: MMV009063, **14**: MMV007591, **15**: MMV665969, **16**: MMV666070, **17**: MMV007224, **18**: MMV665794, **19**: MMV666057, and **20**: MMV665799 were purchased from Specs (Delft, Netherlands), and MolPort (Riga, Latvia). Praziquantel was purchased from Sigma-Aldrich (Buchs, Switzerland) GmbH. Compounds **1–20** were dissolved in DMSO for drug stock solutions of 10 mg/ml for *in vitro* evaluations. Culture medium for newly transformed schistosomula (NTS) was made by supplementing Medium 199 (Lubioscience, Lucerne, Switzerland) with 5% heat-inactivated fetal calf serum (iFCS), penicillin (100 U/ml), and streptomycin (100 µg/ml) (Lubioscience, Lucerne, Switzerland). Culture medium for adult worms was prepared by supplementing RPMI 1640 with 5% iFCS, penicillin (100 U/ml), and streptomycin (100 µg/ml).

### Preparation of Newly Transformed Schistosomula (NTS)

*S. mansoni* cercariae (Liberian strain) were harvested from infected intermediate host snails (*Biomphalaria glabrata*) following in-house standard procedures. Collected cercariae were mechanically transformed to NTS as described previously [25], [26]. The obtained NTS suspension was adjusted to a concentration of 100 NTS per 50 µl using supplemented Medium 199. NTS suspensions were incubated (37°C, 5% CO_2_ in ambient air) for a minimum of 12 to 24 hours until usage to ensure completed conversion into schistosomula [27].

### Ethics Statement

*In vivo* studies were conducted at the Swiss TPH, Basel, and approved by the veterinary authorities of the Canton Basel-Stadt (permit no. 2070) based on Swiss cantonal and national regulations. Experimentation at LSHTM was carried out under the UK Animals Scientific Procedures Act 1986 with approval from the LSHTM Ethics committee.

### Maintenance of Mice and Infection with *S. mansoni*

Animals (female NMRI, 3-week old, weight ca. 14 g) were purchased from Charles River (Sulzfeld, Germany) and allowed to adapt under controlled conditions (temperature ca. 22°C; humidity ca. 50%; 12-hour light and dark cycle; free access to rodent diet and water) for one week. Mice were infected by subcutaneous injection with ∼100 *S. mansoni* cercariae each, harvested from infected snails. For *in vitro* studies on adult flukes, schistosomes were collected from the hepatic portal and mesenteric veins of infected mice 7–8 weeks post infection [28]. Freshly harvested schistosomes were placed in supplemented RPMI culture medium, quickly rinsed, and stored at 37°C, 5% CO_2_ until usage.

### *In Vitro* Compound Screening Cascade on *S. mansoni* at Swiss TPH

Initially, all compounds were tested at a concentration of 100 µM on *S. mansoni* NTS. Active compounds progressed into a secondary screening at 33.3 µM. For this purpose drug stock solutions were diluted in 96-flat bottom well plates (BD Falcon, USA) with supplemented Medium 199 and 50 µl of prepared NTS suspension (100 NTS/well) to the desired final concentration of 100 µM or 33.3 µM, respectively. Each drug was tested at least in triplicate and the highest concentration of DMSO served as control. Plates were incubated at 37°C, 5% CO_2_. NTS were evaluated by microscopic readout (Carl Zeiss, Germany, magnification 80–120×) using a viability scale as previously described with regard to death, changes in motility, viability, and morphological alterations 72 hours post drug exposure [25], [26]. To ensure the accuracy of our assay, 45 compounds that lacked activity at one of the tested concentrations, were randomly selected and retested at 33.3 µM. Compounds that killed the NTS at 72 hours after exposure in at least one well were deemed active and selected for further testing.

In the next step, the IC_50_ was determined for active compounds from the preceding screens. Drug dilution series were prepared in 96-flat bottom well plates with concentrations 2.1, 4.2, 8.4, 16.7, and 33.3 µM using supplemented culture medium. The prepared NTS suspension was then added to each well and plates were incubated at 37°C, 5% CO_2_. NTS incubated in the presence of the highest DMSO concentration and praziquantel served as control. Drug effects on NTS were evaluated 72 hours post exposure, using a viability scale, as described above. Each concentration was tested in duplicate and experiments were repeated once.

Compounds presenting IC_50_ values ≤10 µM were then tested at a concentration of 33.3 µM on adult worms in duplicate. Drug stock solutions (10 mM) were diluted in supplemented RPMI 1640 culture medium reaching a final concentration of 33.3 µM in 24-flat bottom well plates (BD Falcon, USA) within a final volume of 2.4 ml. At least three schistosomes of both sexes were added to each well. Schistosomes incubated in the presence of the highest concentration of DMSO served as control. Plates were incubated for 72 hours at 37°C, 5% CO_2_. Seventy-two hours post drug exposure *S. mansoni* were examined phenotypically by microscope using the motility scale described before [29]. Drugs leading to the death of schistosomes 72 hours post exposure were characterized further and their IC_50_ (IC_90_) values were determined. Specifically, drug dilution series were prepared in 24-flat bottom well plates (BD Falcon, USA) with concentrations of 0.31, 0.93, 2.78, 8.33, and 25.0 µg/ml using supplemented RPMI culture medium and freshly prepared drug stock solutions (10 mg/ml). At least three schistosomes of both sexes were added to each well and plates were incubated at 37°C, 5% CO_2_. Parasites incubated in the highest DMSO concentration and praziquantel served as controls. Drug effects were evaluated 72 hours post exposure as described above. Each concentration was tested in duplicate and trials were repeated once.

### *In Vitro* Screening on Adult Schistosomes at LSHTM

Adult worm drug testing was performed as previously reported [29] with some modifications as described. Worms of a Puerto Rican strain of *S. mansoni* were obtained by portal perfusion of CD1 mice (Charles River, UK) 6 weeks post-infection. Three pairs of worms were added to the wells of 48-well plates (Nunc, UK) in 1 ml complete DMEM medium supplemented with 10% fetal calf serum, 2 mM L-glutamine, 100 U/ml penicillin, and 100 µg/ml streptomycin (cDMEM). Compounds were tested at 15 µM containing 0.15% DMSO in single wells. Negative controls contained worms cultured in cDMEM alone and in cDMEM with 0.15% DMSO. Positive control wells contained worms cultured in praziquantel (Sigma-Aldrich, UK) at 10 µM. Cultures were incubated at 37°C and 5% CO_2_. Effects were assessed on day 5 of culture using an inverted microscope (Leitz Diavert Wetzlar, Germany). Any compounds producing complete immotility or ≥70% worm motility inhibition plus severe morphological damage were considered hits in the primary screen [29]. Active compounds were then tested for IC_50_ value determination at a concentration range from 0.55–15 µM in single wells.

### *In Vitro* Characterization of Lead Candidates on Adult Schistosomes

The onset of action (length of time needed before an antischistosomal effect was visible) was determined for selected compounds *in vitro* by evaluating the IC50 at a time-range of 1–72 hours (1, 2, 4, 7, 10, 24, 48, and 72 hours) post drug exposure, as described above. The onset of action of praziquantel was also studied. Additionally, possible protein binding effects were studied for three lead candidates and praziquantel. For that purpose RPMI medium was supplemented with two different iFCS concentrations (0% and 50%) and IC50 values were calculated for the different conditions. Furthermore, IC50 values were determined after varying drug exposure times (1, 2, or 4 hours) followed by incubation in drug free RPMI medium for 72 hours.

### *In Vivo* Screening Using the Chronic *S. mansoni* Mouse Model

Groups of 3–4 NMRI mice characterized by a patent *S. mansoni* infection (49 days post-infection) were treated orally with the test drug using either single oral doses of 400 mg/kg or 80 mg/kg administered on four consecutive days. An additional dosage regimen of 100 mg/kg administered four times every 4 hours was tested for the 2 most active compounds (**2**, **17**). Compounds were freshly prepared in an aqueous hydroxypropyl methyl cellulose (HPMC) (1%): DMSO (95∶5) formulation. Eight to sixteen untreated mice served as controls. Fourteen days post-treatment animals were killed by the CO_2_ method and were dissected and the worms were sexed and counted [28]. Mean worm burdens of treated mice were compared to the mean worm burden of untreated animals and worm burden reductions were calculated.

### Statistics

Parasite viability values of NTS and adult schistosomes obtained from microscopic evaluation were averaged (means (+/− standard deviation)) using Microsoft Excel. IC50 and IC90 values of test compounds were determined using the CompuSyn software (Version 3.0.1, 2007; ComboSyn Inc., USA) and Microsoft XLfit version 5.1.0.0 (2006–2008 ID Business Solutions Ltd). Selectivity indices were calculated by dividing the IC50 of the MRC-5 cells-fibroblast cytotoxicity data by the IC50 of the adult worm assay. The Kruskal-Wallis test was applied for *in vivo* studies, comparing the worm burden of the treated animals and control animal groups. A difference in worm burden was considered to be significant at a significance level of 5% (StatsDirect, version 2.7.2.; StatsDirect Ltd., UK).

## Results

### *In Vitro* Activity Determined on NTS and Adult Schistosomes at Swiss TPH

**Figure 1:**
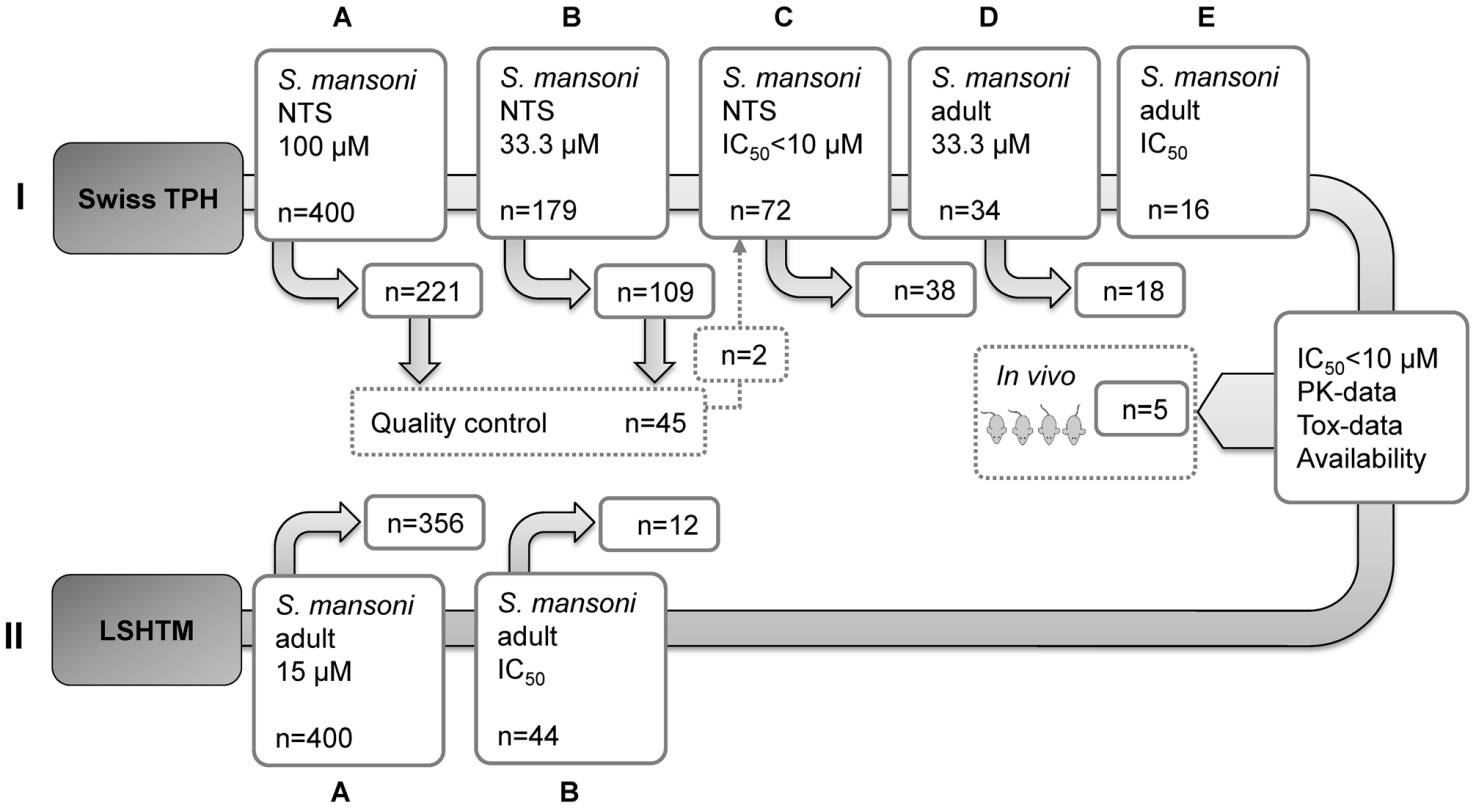
Screening flow. Screening was conducted at the Swiss TPH (screening cascade **I**; steps **A**–**E** and Quality control): Primary screening steps (yes/no filters) of 100 µM and 33.3 µM resulted in 179 and 72 hits, respectively. Active compounds (n = 72) moved on to Step **C** and IC_50_ values were evaluated on NTS. Thirty-four compounds showed activities with IC_50_ values <10 µM and pre-screening was conducted on adult schistosomes (Step **D**). Active compounds (n = 16) with schistosomicidal effects at 33 µM compound concentration were further characterized (step **E**). The quality control represents randomly selected compounds from compounds classified as non-active from the pre-screening steps (step **A**/**B**) on NTS which were re-evaluated in step **B**. In parallel, all compounds (n = 400) were studied at the LSHTM in London (screening cascade **II**): step **A**, all 400 compounds were screened on *S. mansoni* adults at 15 µM. Step **B: 44** compounds were active and these were then tested for IC_50_ determination on adult worms. From both screening cascades, five compounds were selected for *in vivo* testing based on pharmacodynamic and pharmacokinetic properties as well as toxicity.

Exposing schistosomula to the test drugs (n = 400) at a concentration of 100 µM resulted in death of NTS for 45% of the tested compounds (n = 179). Schistosomicidal effects were observed for 18% of these active compounds (n = 72) at the lower concentration of 33.3 µM (Figure 1). A diverse range of chemical scaffolds was observed amongst active compounds. Successful candidates were characterized further on NTS. Promising antischistosomal activity (IC_50_: 1.4–9.5 µM) was observed for 34 compounds, two of which were identified during our quality control re-evaluation of 45 compounds and nine of which showed comparable or increased activity (IC_50_: 1.4–2.4 µM) to praziquantel (IC_50_: 2.2 µM).

All hits (IC_50_<10 µM) (n = 34) were next tested at a concentration of 33.3 µM on adult *S. mansoni*. Seventy-two hours post drug exposure, 16 (**1–16**) of these compounds (Table S1) killed the adult worms. Four of the ten compounds with high activities (IC_50_<2.5 µM) on NTS lacked antischistosomal activity on adult worms. The 16 active candidates were further characterized by IC_50_ value determination. The highest *in vitro* activities were observed for the diaminoquinazoline derivative **1** (IC_50_: 0.8 µM) the diarylurea **2** and diarylamide **3**, presenting IC_50_ values of 0.8 and 1.3 µM, respectively (PZQ: 0.2 µM). IC_50_ values ranging from 2.6–9.2 µM were calculated for compounds **4**–**11**, whereas only moderate activity (IC_50_ values >10 µM) was determined for five compounds (**12–16**). Compounds with IC_50_>10 µM were excluded from further consideration, meaning only eleven compounds were considered as hits.

### *In Vitro* Activity Determined on Adult Schistosomes at LSHTM

Forty-four compounds were classified as hits (compounds producing complete immotility or ≥70% worm motility inhibition plus severe morphological damage) against adult *S. mansoni in vitro* at a concentration of 15 µM. These compounds were further tested for IC_50_ values (Table S1). Twelve compounds showed IC_50_ values >15 µM. Fourteen compounds revealed IC_50_ values between 10–15 µM. Eighteen compounds had IC_50_ values <10 µM. To provide a comparison with the Swiss TPH assays, the 32 hits were subsequently tested using the schistosomula assay at LSHTM [30]. This showed generally good concordance with the LSHTM adult assay, in that all adult hits with IC_50_<10 µM were also hits in the larval assay (Table S1).

### Selection of Lead Candidates

**Figure 2:**
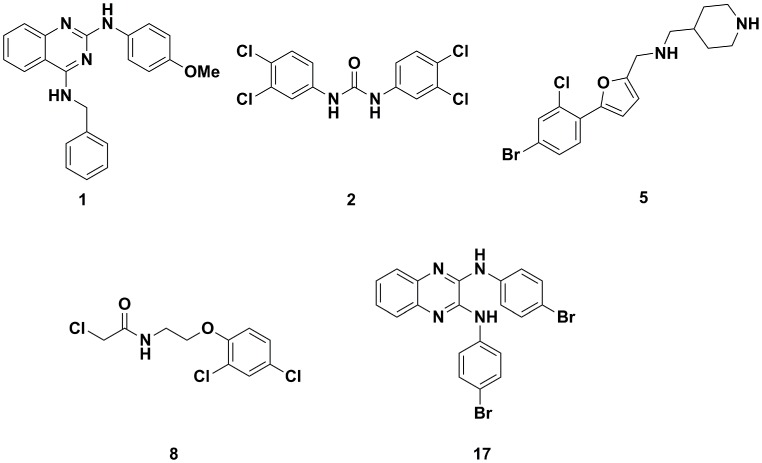
Chemical structures of the five lead compounds selected for *in vivo* studies.

Based on *in vitro* performance on the adult worms (Table S1), toxicity, pharmacokinetic (PK) properties and availability of the compounds, five lead candidates (**1**, **2**, **5**, **8**, **17**) (Figure 2) were selected for *in vivo* testing and in depth characterization *in vitro*. In more detail, 11 compounds were excluded after comparing their IC_50_ values and PK parameters (C_max_, t_max_, t_1/2_, AUC). Four compounds showed poor antischistosomal activity (IC_50_>10 µM) and four compounds showed poor bioavailability (C_max_<IC_50_ of the corresponding compound). Ten compounds were characterized by low selectivity indices (SI<1) and two were not commercially available.

Four active compounds were derivatives belonging to the class of diarylureas and two compounds were characterized as dianilinoquinoxalines. Only the most active candidate of each chemical group, compound **2** and compound **17**, was selected for *in vivo* studies. A summary of the IC_50_ values, toxicity and pharmacokinetic parameters of the lead candidates is provided in Table 1.

**Table 1:** Characterization of five lead candidates selected for *in vivo* testing.

			*P. falciparum* 3D7		NTS		Adult *S. mansoni*		MRC-5 cells-fibroblast		PK- data				
Compound	Molecular weight (g/mol)	ALogP	Inhibition at 5 µM (%)	EC_50_ (nM)	IC_50_ (µM)	R	IC_50_ (µM)	R	IC_50_ (µM)	Selectivity index	Dosage (mg/kg)	C_max_ (µmol/l)	t_max_ (hours)	AUC_0-last_ (h*µM/l)	t_1/2_ (hours)
1	356.42	4.3	-	589	2.7	0.9	0.8	0.9	12.38	15.48	47.8	0.054	7	0.37	>>3
2	350.03	5.2	96	1160	4.7	1	0.8	1	32.00	40.00	46.3	4.4	4.7	30.2	NR
5	383.71	3.9	96	800	1.8	0.9	3.4	0.9	4.03	1.18	50.3	0.37	1.1	1.7	5.2
8	282.55	2.9	94	555	3.4	1	6.3	0.9	16.23	2.58	40.3	0.57	0.3	0.9	2.4
17	470.16	5.7	98	1061	-	-	0.8	-	5.88	7.08	62.5	12.4	8	73	NR

*In vitro* activity on *P. falciparum* 3D7, NTS, adult *S. mansoni*, cytotoxicity on MRC5-cells, and pharmacokinetic parameters* of 5 active compounds selected for *in vivo* studies identified in 2 parallel screens at the Swiss TPH and LSHTM.

PK parameters are unpublished data, *In vitro* activity on *P. falciparum* 3D7 and cytotoxicity on MRC5-cells can be found at http://www.mmv.org/research-development/malaria-box-results.

### *In Vitro* Characterization of Lead Candidates on Adult Schistosomes

**Figure 3:**
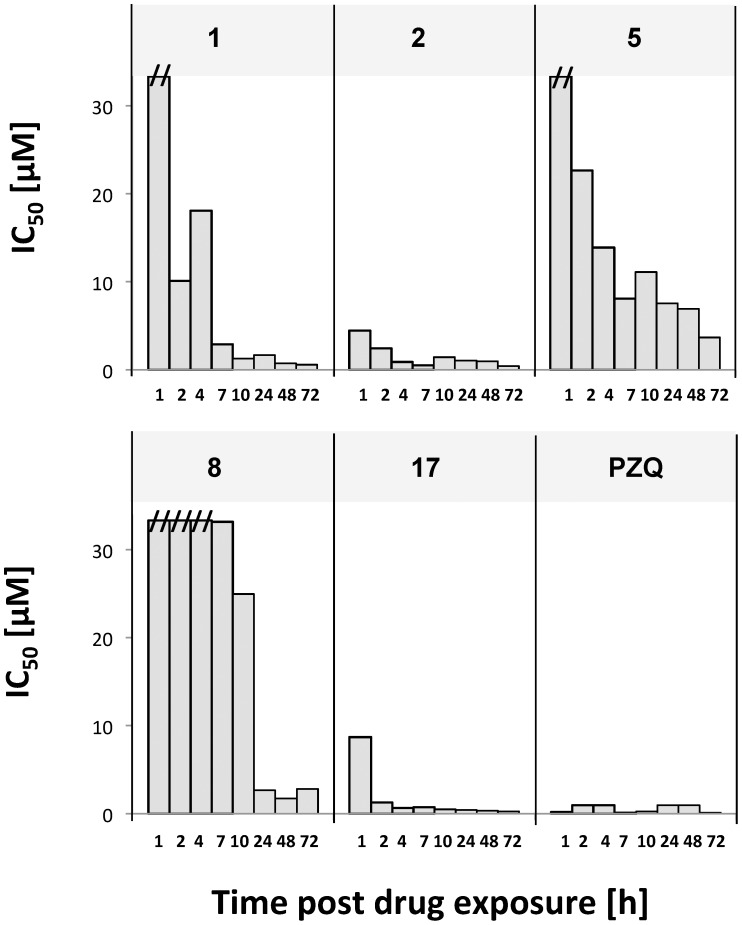
Adult worm IC_50_ values of five *in vivo* candidates over time post drug exposure. Values were determined 1, 2, 4, 7, 10, 24, 48 and 72>33.3 µM are indicated with (**//**). PZQ: praziquantel.

The onset of action was studied in compounds selected for *in vivo* testing (n = 5) and compared to the onset of action for praziquantel (Figure 3). Compound **2** was the fastest acting drug, presenting an IC_50_<5 µM already after 1 hour of *in vitro* exposure, followed by compound **17** with an IC_50_<10 µM, 1 hour post incubation. Compound **1** was intermediate in speed with an onset time of 7 hours post-incubation. Compound **8** had fully exerted its antischistosomal properties 24 hours following incubation, while compound **5** was slow acting (exposing its full antischistosomal activity only 72 hours post treatment). In comparison, praziquantel exposed its entire antischistosomal activity already after 1 hour of drug exposure (IC_50_: 0.2 µM).

The determined IC_90_ values of the lead candidates were 2–5 fold higher than the observed IC_50_ values 72 hours post exposure and thus the concentration-response curves for these compounds are quite steep (Table 2). Comparatively, praziquantel even showed a 13-fold difference between the two values. Praziquantel lead very quickly to a strong motility inhibition and morphological changes, whereas higher concentrations (IC_90_: 2.0 µM) were necessary to actually kill the worms.

**Table 2:** Adult worm IC_50_ and IC_90_ values of five *in vivo* candidates compared to praziquantel 72 hours post drug exposure.

Compound	1	2	5	8	17	PZQ
**IC_50_ (µM)**	0.6	0.5	3.7	2.8	0.3	0.2
**IC_90_ (µM)**	1.7	2.2	9.9	7.9	1.2	2.0
**Ratio IC_90_/IC_50_**	2.8	4.8	2.7	2.8	4.1	13.1

PZQ: Praziquantel.

### *In Vivo* Findings

Compound **2** and **17** revealed the highest *in vivo* activity with worm burden reductions (WBR) of 52.5% (dosage 1×400 mg/kg; p<0.005) and 53.4% (dosage 4×100 mg/kg; p<0.005), respectively (Table 3). In addition, both treatment regimens using multiple doses of compound **2** resulted in significant worm burden reductions of 46.0% (4×80 mg/kg; p<0.005) and 31.2% (4×100 mg/kg; p<0.05). Treatment with a single 400 mg/kg dose of compound **17** resulted in a significant worm burden reduction of 40.8% (p<0.05), while multiple treatment courses of 80 mg/kg over four consecutive days achieved a lower effect (WBR: 25.5%, p<0.05). Compounds **1**, **5**, and **8** lacked *in vivo* activity (WBR 0–18.7%). No significant differences were observed between total and female worm burden reductions.

**Table 3:** Worm burden reductions observed for the five lead candidates in *S. mansoni* infected mice.

Compound	Dosage	Mice(n)	Total worms recovered (n)	SD	WBR (%)	Control batch
**Control 1**	-	16	38.5	13.2	-	-
**Control 2**	-	9	40.4	13.5	-	-
**Control 3**	-	8	35.4	13.8	-	**-**
**1**	1×400 mg/kg	4	50.3	12.7	0	1
	4×80 mg/kg	3	34.7	14.2	9.9	1
**2**	1×400 mg/kg	4	18.3	5.1	52.5**	1
	4×80 mg/kg	4	20.8	6.1	46.0**	1
	4×100 mg/kg	4	27.8	7.0	31.2*	2
**5**	1×400 mg/kg	4	37.8	8.1	1.8	1
	4×80 mg/kg	4	33.5	16.9	12.7	1
**8**	1×400 mg/kg	3	31.3	6.5	18.7	1
	4×80 mg/kg	3	31.7	8.5	17.7	1
**17**	1×400 mg/kg	4	22.8	10.9	40.8*	1
	4×80 mg/kg	3	28.7	10.1	25.5*	1
	4×100 mg/kg	4	16.5	8.5	53.4**	3

Mice harbored a patent *S. mansoni* infection. Different dosage regimens were used (1×400 mg/kg, 4×80 mg/kg on four consecutive days or 4×100 mg/kg every 4 hours).

WBR: Worm burden reduction.

p-value<0.05.

p-value<0.005.

### Protein-Binding and Short-Term Drug Exposure of Leads

Compound **17** showed a 7-fold increase in activity in iFCS-free medium (IC_50_: 0.3 µM) versus incubation in 50% serum supplemented medium (IC_50_: 2.1 µM) (Table S2). A strong increase in activity in serum free medium was observed for praziquantel (IC_50_: 0.02 µM). No altered activities were detected for compound **2** within varying iFCS-concentrations. Short-term exposure of schistosomes to compound **2** or praziquantel (1–4 hours) followed by incubation in drug free medium for 72 hours resulted in high IC_50_ values, ranging from 51.1 µM (1 hour) to 24.6 µM (4 hours) for compound **2** and from 96.1 µM to 7.7 µM for praziquantel (Figure S3). These values are much higher than the IC_50_ values determined when the worms are continuously exposed to the drugs for 72 hours (**2**: IC_50_: 0.8 µM; PZQ: IC_50_: 0.2 µM). Incubation of schistosomes for 4 hours with compound **17** achieved similar effects (IC_50_: 1.3 µM) (Figure S3) as described for the 72 hours exposure time (IC_50_: 0.8 µM) (Table S2).

## Discussion

The aim of this study was to investigate the antischistosomal potential of 200 drug-like and 200 probe-like compounds assembled in the MMV Malaria Box. The MMV Malaria Box provided a unique opportunity: commercially available compounds with confirmed *in vitro* activity against *P. falciparum* serve as good starting material for antischistosomal R&D, as many antimalarials have antischistosomal activity [16], [23], [31]. In addition, and in line with the target characteristics of a trematocidal lead candidate [20], properties of the drug-like compounds are commensurate with oral absorption and the presence of known toxicophores is minimized.

NTS were used as a prescreening tool at Swiss TPH, since their use greatly reduces the need for laboratory animals and thus is a major contributor to the 3 R rules (replace, reduce, refine) [25]. Nearly half of the tested compounds (45%) presented schistosomicidal effects on the schistosomular stage at a concentration of 100 µM. Given this high hit rate, compounds which were not lethal on NTS did not progress further. This might be a limitation of the Swiss TPH screening, since many effective anthelmintics (including praziquantel at low concentrations) cause paralysis rather than death of worms [32]. Thirty-four of the active compounds had IC_50_ values ranging from 1.4 to 9.5 µM, suggesting that both parasites, *P. falciparum* and *S. mansoni*, have a similar drug sensitivity profile. About half of the compounds active against NTS (n = 16) revealed good to moderate activity on the adult stage (IC_50_: 0.8–22.3 µM). Several compounds that showed high antischistosomal effects on schistosomula lacked activity on adults. This phenomenon, where the hit rates were higher against the larval stages than against the adult stages, has been previously reported [14], [33]. A higher sensitivity of the larval stage, or mode of action dependent effects might partially explain this higher hit rate: for example recent studies with various peroxide classes documented less activity on the adult stage than on the NTS stage [15].

The parallel screening at LSHTM screened all compounds directly on adult schistosomes. Thirteen additional compounds active against adult worms (IC_50_<10 µM) were identified at LSHTM. Nine of these lacked activity against NTS at Swiss TPH (Figure S2). Interestingly, these compounds showed activity against NTS at LSHTM (Table S1). On the other hand, four compounds with activity (IC_50_<10 µM) against NTS and adult worms identified at Swiss TPH lacked activity in the LSHTM screen. Overall, 22 compounds had an IC_50_<10 µM against adult worms in at least one of the screens. Only five compounds were characterized by an IC_50_<10 µM in both screenings. Strain differences but also different ways of assay set up and readout might offer an explanation for these results. Nonetheless, follow up studies to clarify these issues are warranted.

Compound **2**, a diarylurea, revealed the highest activity against adult *S. mansoni in vitro*. In addition, our onset of action studies revealed that it was the fastest acting compound, comparable to praziquantel. The compound is characterized by an intriguingly simple chemistry and can be easily synthesized. The class of N,N′-diarylureas was recently found to activate heme-regulated inhibitor kinase which inhibits translation initiation and plays a central role in cancer initiation [34]. Additionally various N,N′-diarylureas, including compound **2**, have been investigated as potential anti-cancer agents and were proposed as promising lead compounds [35]. Significant worm burden reductions of 52.5%, 46.0%, and 31.2% were observed with compound **2** following single oral dosing with 400 mg/kg, 80 mg/kg on four consecutive days and 4×100 mg/kg every four hours, respectively. This might indicate that *in vivo* activity follows a time over threshold model rather than it being C_max_ driven. However, based on the *in vitro* performance and pharmacokinetic data, a better *in vivo* outcome was expected. Our follow-up *in vitro* studies, which studied protein binding and the short-term drug exposure, might offer an explanation for this discrepancy. Short incubation times (1 to 4 hours) were not sufficient to kill the worms, since most of the parasites recovered 3 days later. Note that compound **2** is characterized by a half-life (t_1/2_) of 4.7 hours and C_max_ of 4.4 µM at 46.3 mg/kg (*po*).

Additionally compound **17**, a 2,3-dianilinoquinoxaline derivative, showed high *in vitro* (IC_50_: 0.83 µM) and significant *in vivo* activity with WBRs between 53.4% (multiple *po* dose of 100 mg/kg every four hours) and 40.8% (single *po* dose 400 mg/kg). This series has been reported to show antimycobacterial activity [36].

The order of *in vivo* activity of the five selected candidates is in line with the onset of action observed *in vitro*. The fastest acting compound **2** exhibited the highest activity *in vivo* followed by compound **17** (WBR: 40.8%). The discrepancy of excellent *in vitro* performance of compound **17**, but only moderate *in vivo* activity might be explained by protein binding effects. Increased activities were observed when incubated sans serum proteins *in vitro*. Notably, short-term incubation of 4 hours was sufficient to exhibit high antischistosomal effects for both drugs. Compounds **1**, **5**, and **8** acted slower (only 7–10 hours post exposure), and lacked activity *in vivo*. This finding is in line with PK properties of these drugs. Since the half-lives of the compounds are rather short (2.4–5.2 hours) plasma concentrations remain insufficiently long above the IC_50_ values for the slow acting compounds to exert *in vivo* activity.

Since a series of related derivatives was present in the MMV Malaria Box, we carried out an initial structure-activity relationship study by sourcing commercially available near neighbors for compounds **1**, **2**, and **5** (Table S3). Exchanging the phenyl-group of **1** with an ethanol group revealed a stage specific sensitivity with activity on NTS, but lacked schistosomicidal effects on adult worms. The substitution pattern on phenyl-residues of compound **2** influenced activity. For example, exchanging the *para*-chloro to a *para*-fluro on one of the phenyl rings led to a two-fold decrease in activity on NTS. Such subtle changes in activity require further investigation with a larger set given the easy chemical accessibility of derivatives.

In conclusion, by screening the MMV malaria box on *S. mansoni* we underlined the potential of compounds with an antimalarial background on schistosomes. We identified two entirely new chemical scaffolds: the N,N′-diarylurea (**2**) and 2,3-dianilinoquinoxaline derivatives (**17**) with antischistosomal *in vitro* activity in the sub micromolar range and moderate *in vivo* activity. The compounds offer promising drug characteristics such as a good pharmacokinetic profile and low cytotoxic potential. Their easy chemistry simplifies further drug optimization steps and offers an excellent starting point for antischistosomal drug discovery and development.

## Supporting Information

Figure S1 Structures of anthelmintic and antimalarial drugs used against schistosomiasis.
(PPT)

Figure S2 Venn diagram for adult *S. mansoni* hits direct screening on adult schistosomes shown in blue (at LSHTM) or with prior screening on NTS followed by screening on the adult stage presented in red (at Swiss TPH).
(PPT)

Figure S3 Adult worm IC_50_ values of the two lead candidates (**2** and **17**) incubated with the compounds for 1, 2, or 4 hours followed by incubation in compound-free medium for 72 hours.
(PPT)

Table S1 Results for the LSHTM and Swiss TPH *in vitro* adult and larval *S. mansoni* screening.
(DOC)

Table S2 IC_50_ values of compounds **2**, **17** and praziquantel (**PZQ**) in RPMI medium supplemented with 0, 5, or 50% iFCS.
(DOC)

Table S3 *In vitro* performance of selected derivatives of *in vivo* candidates **1**, **2**, and **5**.
(DOC)
