# Supplementary figures and images for: Orally Active Antischistosomal Early Leads Identified from the Open Access Malaria Box
Source: PLoS Negl Trop Dis. 2014 Jan 9;8(1):e2610. doi: 10.1371/journal.pntd.0002610 (PMC3886923; doi:10.1371/journal.pntd.0002610)

## Slide 1
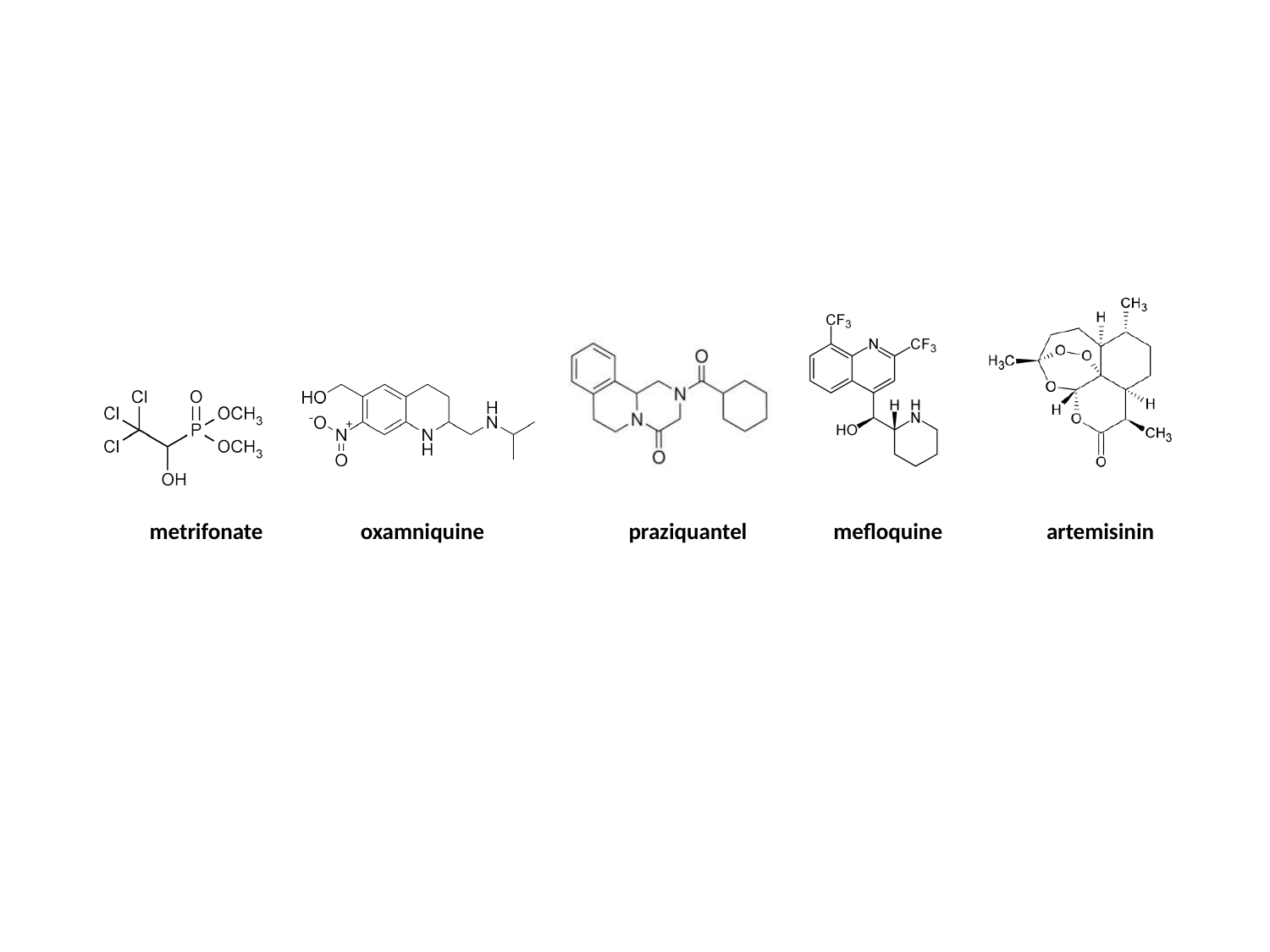

metrifonate
oxamniquine
praziquantel
mefloquine
artemisinin

Supplement: Figure S1 — Structures of anthelmintic and antimalarial drugs used against schistosomiasis. (PPT) [file pntd.0002610.s001.ppt]

## Slide 1
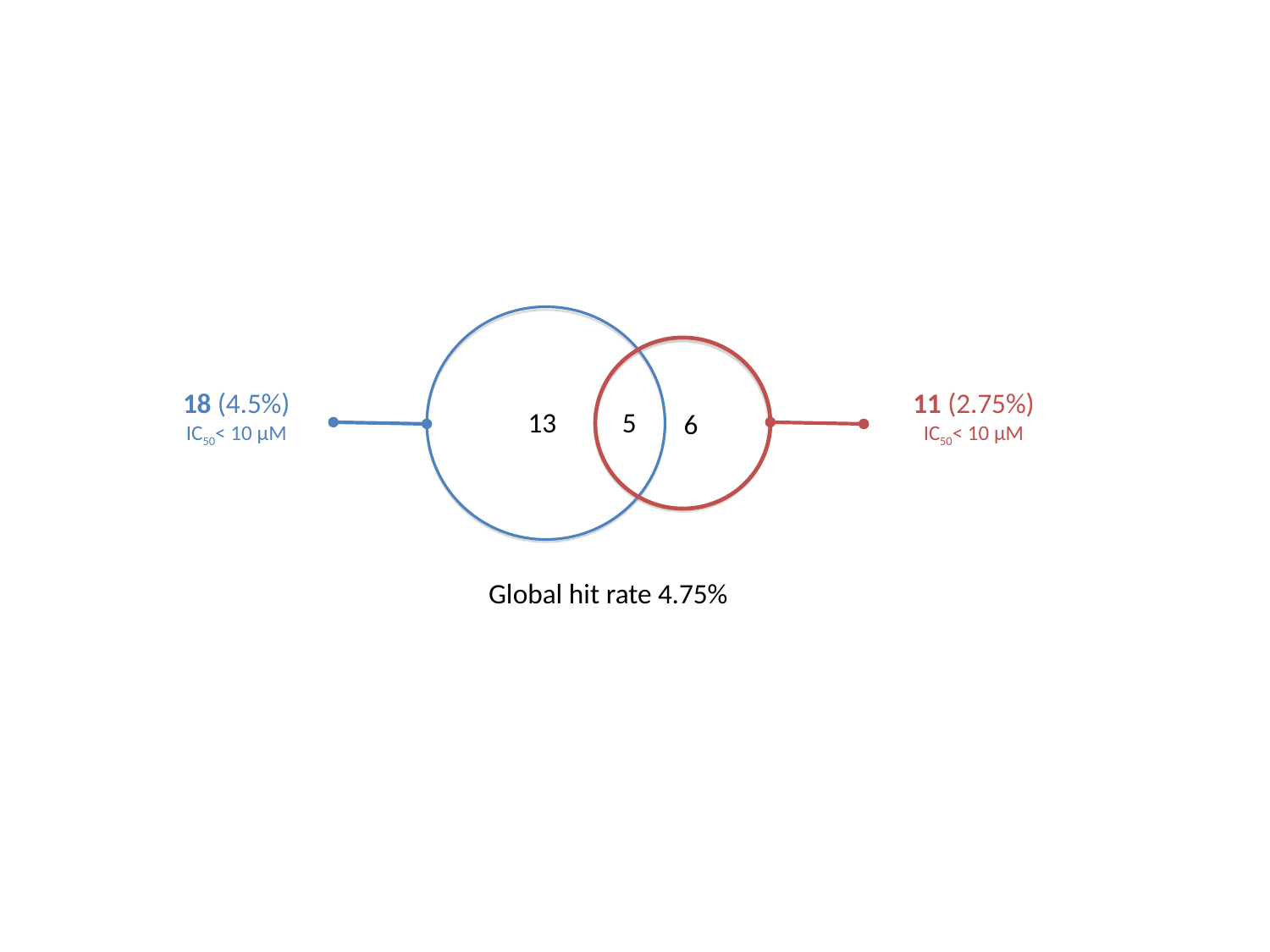

18 (4.5%)
IC50< 10 μM
11 (2.75%)
IC50< 10 μM
13
5
6
Global hit rate 4.75%

Supplement: Figure S2 — Venn diagram for adult S. mansoni hits direct screening on adult schistosomes shown in blue (at LSHTM) or with prior screening on NTS followed by screening on the adult stage presented in red (at Swiss TPH). (PPT) [file pntd.0002610.s002.ppt]

## Slide 1
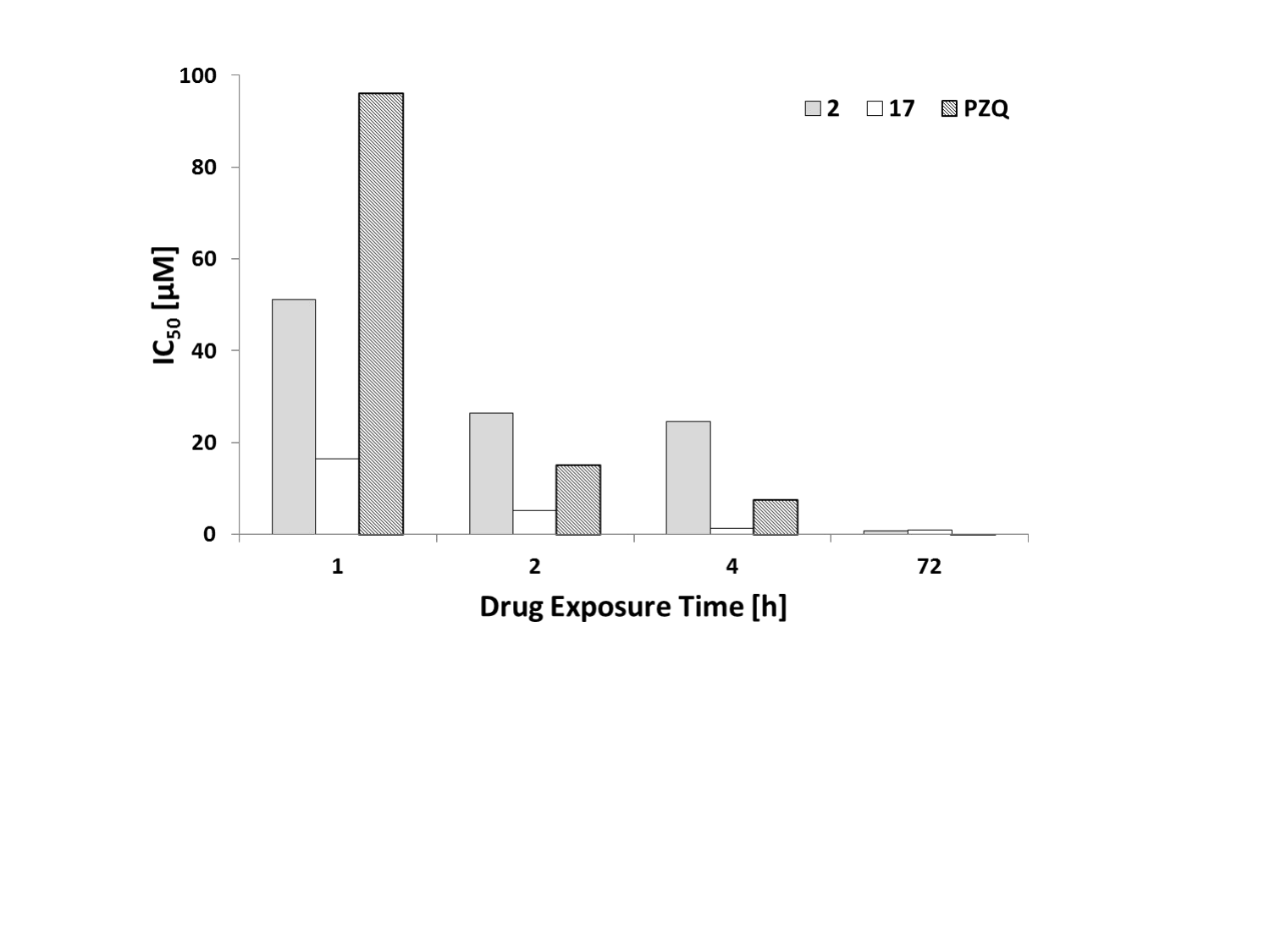

Supplement: Figure S3 — Adult worm IC50 values of the two lead candidates (2 and 17) incubated with the compounds for 1, 2, or 4 hours followed by incubation in compound-free medium for 72 hours. (PPT) [file pntd.0002610.s003.ppt]

**Table S3.**

|  |  | **Effect NTS** | | **Effect Adult** |  |  |  | **Effect NTS** | | **Effect Adult** |
| --- | --- | --- | --- | --- | --- | --- | --- | --- | --- | --- |
|  |  | **33.3 µM**  **(%)** | **IC50**  **(µM)** | **33.3 µM**  **(%)** |  |  |  | **33.3 µM**  **(%)** | **IC50 (µM)** | **33.3 µM**  **(%)** |
| **1** | **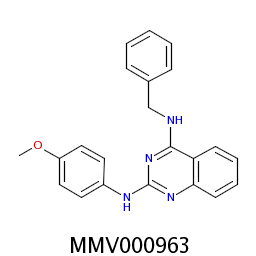** | 100 | 2.7 | 100 |  | **2** | **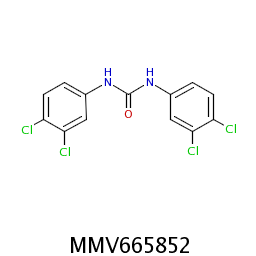** | 100 | 4.7 | 100 |
| **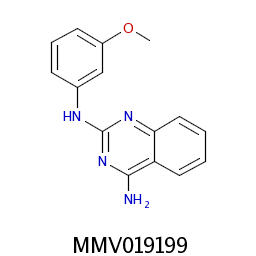** | 17 | - |  |  | **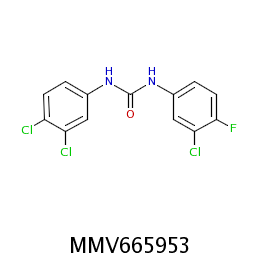** | 100 | 11.3 |  |
| **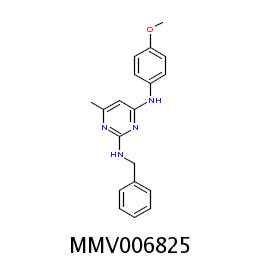** | 42 | - |  |  | **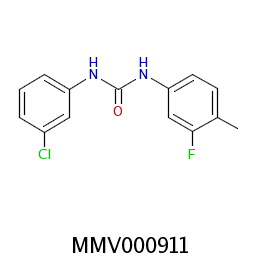** | 100 | 6.2 | - |
| **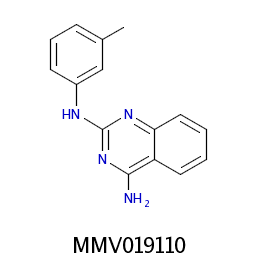** | 67 | - |  |  | **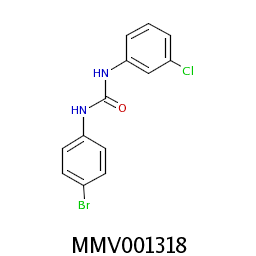** | - |  |  |
| 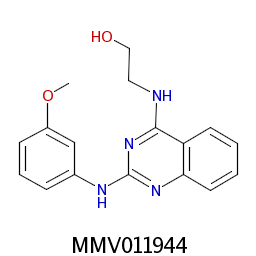 | 100 | 3.8 | 62.5 |  | 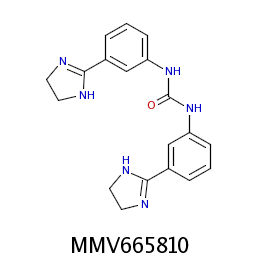 | - |  |  |
| 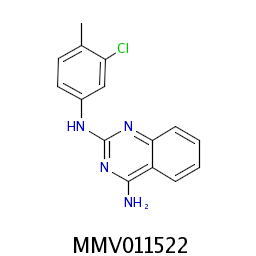 | - |  |  |  | **5** | **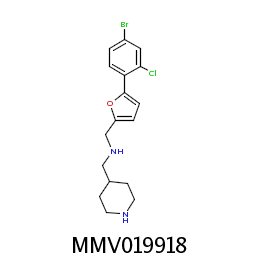** | 100 | 1.8 | 100 |
| 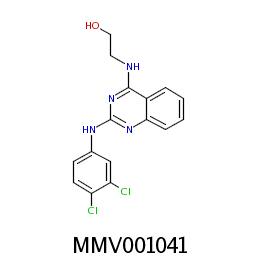 | 67 |  |  |  | **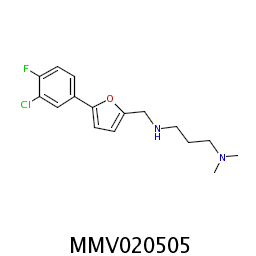** | 100 | 2.8 | - |
| 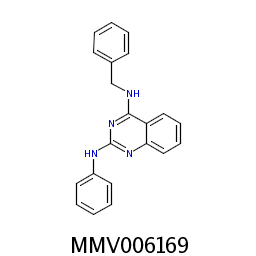 | 67 |  |  |  | **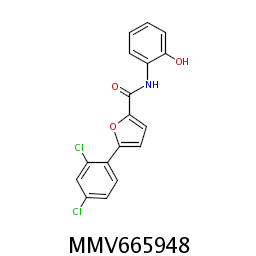** | 31 | - |  |
| 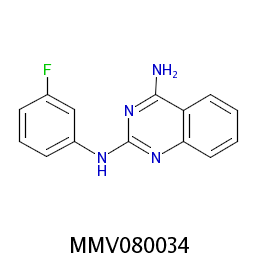 | 0 |  |  |  | **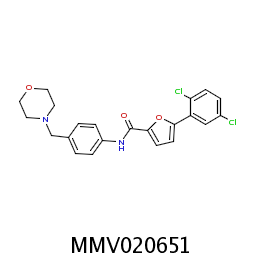** | 100 | >10 |  |

Supplement: Table S3 — In vitro performance of selected derivatives of in vivo candidates 1, 2, and 5. (DOC) [file pntd.0002610.s006.doc]
