# Supplementary material for: Orally Active Antischistosomal Early Leads Identified from the Open Access Malaria Box
Source: PLoS Negl Trop Dis. 2014 Jan 9;8(1):e2610. doi: 10.1371/journal.pntd.0002610 (PMC3886923; doi:10.1371/journal.pntd.0002610)
Supplement: Table S1 — Results for the LSHTM and Swiss TPH in vitro adult and larval S. mansoni screening. (DOC) [file pntd.0002610.s004.doc]

**Table S1.**

| **Compound ID** | **Manuscript ID** | **Swiss TPH**  **NTS IC_50_ (μM)** | **LSHTM**  **NTS (15 μM)** | **Swiss TPH**  **Adult worm IC_50_ (μM)** | **LSHTM**  **Adult worm IC_50_ (μM)** |
| --- | --- | --- | --- | --- | --- |
| MMV000963 | **1** | 2.7 | Hit | 0.8 | 14.8 |
| MMV665852 | **2** | 4.7 | Hit | 0.8 | 0.8 |
| MMV665807 | **3** | 1.8 | Hit | 1.3 | 0.6 |
| MMV019555 | **4** | 2.3 | - | 2.6 | >15* |
| MMV019918 | **5** | 1.8 | Hit | 3.4 | 9.7 |
| MMV000445 | **6** | 2.9 | Hit | 5.1 | 5.2 |
| MMV019780 | **7** | 2.3 | Hit | 6.1 | 14.8 |
| MMV665927 | **8** | 3.4 | - | 6.3 | >15* |
| MMV665941 | **9** | 3.6 | Hit | 8.5 | 9.7 |
| MMV000634 | **10** | 2.7 | - | 8.9 | >15* |
| MMV665830 | **11** | 2.8 | - | 9.2 | >15* |
| MMV666054 | **12** | 1.9 | Non-Hit | 11.2 | 13.4 |
| MMV009063 | **13** | 5.3 | - | 11.4 | >15* |
| MMV007591 | **14** | 2.0 | - | 13.9 | >15* |
| MMV665969 | **15** | 2.6 | - | 18.0 | >15* |
| MMV666070 | **16** | 3.1 | - | 22.3 | >15* |
| MMV007224 | **17** | >33.3 | Hit | - | 0.8 |
| MMV665794 | **18** | >33.3 | Hit | - | 1.1 |
| MMV666057 | **19** | >33.3 | Hit | - | 14.7 |
| MMV665799 | **20** | >100 | Non-Hit | - | 14.8 |
| MMV665953 | **21** | >100 | Hit | - | 1.0 |
| MMV001318 | **22** | >33.3 | Hit | - | 3.6 |
| MMV000788 | **23** | 1.4 | Hit | >33.3 | 4.4 |
| MMV007384 | **24** | 16.9 | Hit | - | 5.0 |
| MMV007181 | **25** | 2.6 | Hit | >33.3 | 6.1 |
| MMV666103 | **26** | >100 | Hit | - | 6.4 |
| MMV000911 | **27** | 4.8 | Hit | >33.3 | 6.5 |
| MMV666022 | **28** | >33.3 | Hit | - | 7.3 |
| MMV665987 | **29** | >33.3 | Hit | - | 9.7 |
| MMV667491 | **30** | 3.6 | Hit | >33.3 | 9.7 |
| MMV006169 | **31** | >33.3 | Hit | - | 9.7 |
| MMV665994 | **32** | >100 | Non-Hit | - | 10.6 |
| MMV396744 | **33** | >100 | Non-Hit | - | 13.1 |
| MMV665883 | **34** | >100 | Non-Hit | - | 13.9 |
| MMV000248 | **35** | 12.6 | Hit | - | 14.2 |
| MMV000444 | **36** | >33.3 | Non-Hit | - | 14.3 |
| MMV666691 | **37** | >33.3 | Non-Hit | - | 14.5 |
| MMV396669 | **38** | 3.7 | Hit | >33.3 | 14.7 |
| MMV006389 | **39** | >100 | Hit | - | 14.7 |
| MMV665939 | **40** | >100 | Non-Hit | - | 14.7 |

* Negative in the LSHTM primary adult assay at 15 µM
