# Supplementary material for: Orally Active Antischistosomal Early Leads Identified from the Open Access Malaria Box
Source: PLoS Negl Trop Dis. 2014 Jan 9;8(1):e2610. doi: 10.1371/journal.pntd.0002610 (PMC3886923; doi:10.1371/journal.pntd.0002610)
Supplement: Table S2 — IC50 values of compounds 2, 17 and praziquantel (PZQ) in RPMI medium supplemented with 0, 5, or 50% iFCS. (DOC) [file pntd.0002610.s005.doc]

**Table S2**.

| **Compound** | **iFCS (%)** | **Adult** | |
| --- | --- | --- | --- |
|  |  | **IC_50_ (µM)** | **R** |
| **PZQ** | **0** | 0.02 | 0.7 |
|  | **5*** | 0.2 | 0.9 |
|  | **50** | 0.6 | 0.9 |
| **2** | **0** | 2.1 | 0.9 |
|  | **5*** | 0.8 | 1.0 |
|  | **50** | 2.3 | 0.9 |
| **17** | **0** | 0.3 | 0.9 |
|  | **10**** | 0.8 | - |
|  | **50** | 2.1 | 1.0 |

Standard assay at Swiss TPH* and LSHTM**

PZQ: Praziquantel
